# The Cardiff Medical School

**Published:** 1914-02-21

**Authors:** 


### THE CARDIFF MEDICAL SCHOOL.

Last year we chronicled the liberality of Mr. (now Sir) W. J. Thomas in giving the sum of £30,000 towards the new buildings for the medical school to be started as part of the University College of South Wales and Monmouthshire. On February 12 last matters were advanced a long stage further, for on that date a deputation waited upon the Chancellor of the Exchequer, to whom Colonel Bruce Vaughan was able to announce that an anonymous donor has promised the balance of the £90,000 which the completed buildings will cost. Fortified by this proof that Wales is willing to do its share of self-help in the creation of a new educational medical centre, the deputation, which consisted of a number of exceedingly able and influential leaders of what is best in Welsh life and thought, urged the Chancellor to grant assistance from the Treasury towards the upkeep of the school.

To this request the Chancellor's answer was not unfavourable. As in duty bound, he reminded the deputation that the Treasury would require to be satisfied as to various points in the organisation of the teaching staff, the equipment of the buildings, and so forth. For this purpose he suggested that an expert committee might be useful to guide him in forming conclusions on the subject. With this proviso he was prepared to say that if Wales would "come forward with like generosity and help to staff the school, he would advise the Government to make a substantial contribution." Exactly what Mr. Lloyd George meant by this is not absolutely clear; but the most natural meaning seems to be that if Wales will raise another £90,000 for the endowment of the school, the Treasury will grant some unspecified amount annually to help out the scheme. However, the precise interpretation of this qualified promise does not concern us now. The arresting fact is that the Cardiff Medical School is evolving, and will soon come into existence as one where students may take a complete course of medical studies right up to graduation. Hitherto they have been able at the most to study three-fifths of the curriculum in Cardiff, and have been obliged to go to London, Edinburgh, Manchester, or elsewhere in order to complete their clinical studies.

This development of medical education in Cardiff will certainly be of great benefit to the population of Wales, provided that the school is made really efficient and the teaching is first-class from the start. We trust it will be, but strenuous efforts will be necessary to get the right men and to keep them; and there will have to be also a ruthless rejection of any members of the staff who fail to reach the front rank in the art of teaching students. In this connection the controversy of last year over the qualifications to be required of the honorary staff of King Edward VII.'s Hospital, Cardiff, will be fresh in the minds of our readers (The Hospital, November 15, 1913, p. 156.

By a narrow majority a proposal was defeated, the object of which was to raise the standard of examinational powers for all future elections. We still think that this was a mistake in policy, though it is quite possible that it may not prove detrimental to the school or the hospital. In our judgment it is of supreme importance that all appointments to the teaching staff of the school and to the visiting staff of the hospital shall be entirely free from that provincialism which has so often cursed Wales in the past. The best men must be secured, irrespective of whether they be English, Scottish, Irish, or Welsh. Thus, and thus only, can the new school hope to prosper.

